# Managing Risk Aversion for Low-Carbon Supply Chains with Emission Abatement Outsourcing

**DOI:** 10.3390/ijerph15020367

**Published:** 2018-02-21

**Authors:** Qinpeng Wang, Longfei He

**Affiliations:** 1 School of Management Science and Engineering, Hebei University of Economics and Business, Shijiazhuang 050061, China; qinpeng@heuet.edu.cn; 2 College of Management and Economics, Tianjin University, Tianjin 300072, China

**Keywords:** carbon efficient operations, emission-reduction outsourcing, low-carbon preference, risk aversion, supply chain coordination

## Abstract

Reducing carbon emissions, including emission abatement outsourcing at the supply-chain level, is becoming a significant but challenging problem in practice. Confronting this challenge, we therefore break down the practice to focus on a low-carbon supply chain consisting of one supplier, one manufacturer and one third-party emission-reducing contractor. The contractor offers a carbon reduction service to the manufacturer. In view of the increasing proportion of Greenhouse Gases (GHG) emissions and absence of carbon reduction policies in developing countries, we adopt the prospect of consumers’ low-carbon preferences to capture the demand sensitivity on carbon emission. By exploiting the Mean-Variance (MV) model, we develop a supply chain game model considering risk aversion. Comparing the supply chain performances of the cases under risk neutrality and risk aversion, we investigate the impact of the risk aversion of the supplier and the manufacturer on the low-carbon supply chain performances, respectively. We show that the risk aversion of chain members will not influence the relationship underlain by the profit-sharing contract between the manufacturer and contractor, whereas they may extend the supplier’s concerning range. Although the manufacturer’s risk aversion has a positive impact on the wholesale price, interestingly, the supplier’s impact on the wholesale price is negative. Furthermore, we propose a contract to coordinate the risk-averse low-carbon supply chain by tuning the aversion levels of the supplier and the manufacturer, respectively. Through numerical study, we draw on managerial insights for industrial practitioners to adopt a low carbon strategy potentially by managing the risk attitudes along the supply chain channel.

## 1. Introduction

Controlling carbon emissions that result in climate change and environment issues is gradually becoming a global challenge urgently needing to be solved, which has been a wide consensus for both academia and industry. Roughly speaking, after realizing industrial and commercial activities among significant sources of carbon emissions, governments around the world have promulgated various regulative policies targeting the control and reduction of carbon emission. Accordingly, the substantial influence of regulations drives companies, particularly those in developing countries, to emphasize emission control for confronting pressure and incidentally attaining long-term success by synthesizing aspects of economic, environmental and societal sustainability. The purpose of this study is to help people understand how firm-level carbon emission can be affected by interactions of supply chain members considering risk attitude in the low-carbon environment.

Stressing on carbon reduction, many transnational companies scheme and voice their own targeted plans of carbon emissions abatement. For instance, Nestlé and Sainsbury’s cooperate with its stakeholders across milk value chain to focus on the impact of commercial activities on environment [1], reducing carbon footprint per cup of coffee by 20.7% in 2013. Furthermore, being different from firm-level carbon-reduction endeavor, inter-firm cooperation within the supply chain shows a significant impact on implementing environmental initiatives [2]. That is because the alignment of chain members is considerable for carbon-reduction but hard to achieve since the complicated carbon-reduction process typically involves manufacturing, product packaging, refrigeration, transportation and even waste disposal. As the interests of the aforementioned links may usually conflict with each other, pursuing self-interest optimization usually causes inefficiency of utilizing resources during this process.

On the other hand, there may emerge some new division of labor in the low-carbon environment. Compared with specialized emission-reduction companies, manufacturing firms often either lack specific emission-reducing technologies or they do it in a costly manner due to disadvantages in resource endowment. This situation offers third-party emission-reducing contractor opportunities to provide carbon reduction for entire supply chains.

As we observed from today’s market, the low-carbon footprint can bring substantially positive effect to promotion and sales amount, such as expanding brand popularity, diminishing the pressure and attracting more customer stream. Consumers with environmental awareness would prefer low-carbon products, generating a positive environmental externality. At this point, some extant studies that examine consumers’ perception show that a low-carbon product has advantages over high-carbon, which is exactly what we intend to study in this paper. The Nielsen Global Survey on Corporate Social Responsibility reports that half of all global consumers in the survey were willing to pay more for goods and services from socially responsible companies. Similarly, Aldy, Kotchen [3] concludes that the average US citizen was willing to pay 13% more for clean electricity [3]. Echeverría et al. obtain that Chilean consumers were willing to pay fluid milk and bread with low carbon footprint 29% and 10% more than their average prices, respectively [4]. The consumer preference for low-carbon goods spurs companies to reduce carbon emissions voluntarily. Similarly, Tait et al. find that consumers from emerging economies have higher willingness to pay for environmentally sustainable lamb than those from the developed world [5].

Although low-carbon goods can cater for the preferences of a specific market segment, it may more likely incur demand risk for supply chain firms due to the uncertainties resulting from the low-carbon market, technology and regulation policies. Hence, it is naturally necessary to consider a firm’s risk tendency by maximizing its utility rather than profit when optimizing decisions of supply chain operations. We can find the risk factors arising in reality, for example, in the context of The Belt and Road Initiative of China. Various firms joining the program from different countries may easily suffer the risk aversion due to cultural conflicts. In this sense, studying risk aversion management within a low-carbon supply chain is imperative since different levels of risk might usually form potential conflicts among supply chain members. Naturally, one may ask here how the risk aversion affects the interactive decisions of the low-carbon supply chain. In this study, we devise two types of carbon-reduction schemes to echo this question, i.e. the customizing and the ordering cases. In the customizing case, the manufacturer firstly determines the payment level for the carbon-reduction service provided by the contractor, which is followed by the contractor’s succeeding carbon-reduction level decision. By contrast, in the ordering case, the contractor moves first to charge the unit-carbon-reduction price followed by the manufacturer’s ordering decision on carbon reduction. Finally, this study intends to demonstrate how chain members’ role reversal of decision-making on pricing and carbon-reducing impacts supply chain performance as well as emission reduction.

Additionally, clarifying the influence of risk attitudes about demand volatility on decision makers is also essential for comprehending the operation mechanism for low-carbon supply chains. Being similar to the well-known double marginalization in traditional supply chain research, individual rationality actuates chain members to opt for their own risk aversion instead of the global interest. However, the risk aversion level that reflects individual judgment is usually neglected and disregarded. Hence, focusing on coordinating the risk-averse supply chain reflects our emphasis on the factor of risk aversion significantly embedded in daily operations.

The remainder of the paper is organized as follows. In Section 2, we start to summarize the extant literature in both fields of the carbon efficient supply chain and mean-variance risk analysis. Section 3 illustrates the notation system, assumptions and preliminary models to be deployed in the study. In Section 4, we address, model and analyze the supply chain setting where the supplier and the manufacturer are both risk neutral during the decision process. In the subsequent Section 5, we analyze the centralized and also the decentralized supply chains, respectively, with the supplier and the manufacturer both risk-averse in each scenario. In Section 6, we devise a contract to coordinate the decentralized risk-averse supply chain. Finally, we conduct a numerical study and present managerial insights in Section 7. The paper comes to the end in Section 8 with concluding remarks and sketches potential research directions in the future.

## 2. Literature Review

Our study highly relates to several streams of literature including carbon emission reduction, outsourcing and risk aversion. As for literature in carbon emission reduction, we refer readers to [6,7] for a comprehensive review of the green supply chain. Surprisingly, carbon-controlling regulations may backfire on the decision-maker’s original intention by shifting production to unregulated regions and increasing global emissions. Similarly, Drake [8] discusses the effect of carbon import tariffs in environments with technology choice and foreign comparative advantage.

There is some literature exploring the reduction of carbon emissions through proper operation strategies in sourcing. Ovchinnikov and Raz [9] investigate the effect of demand uncertainty and consumption externality on governmental capability of coordinating, pricing and supplying public goods with rebates and subsidy. Alhaj et al. [10] analyze the joint location-inventory problem and apply it to reduce carbon emission. There exist other influential studies considering similar settings to reduce carbon emission via typical operations strategies adjustment [2,11,12,13]. Some extant research investigates the way of reducing carbon emission in energy and bio-fuel supply chains [14,15,16]. Although the aforesaid literature considers the impact of governmental regulation on emission-reducing relevant behaviors of firms, they neither analyze the supply chain coordination nor the decision-maker’s risk perception. In contrast, by filling this gap we propose and study the risk-averse supply chains, within which chain members outsource their carbon reduction services from external contractors specializing in emission reduction.

Most of the supply chain outsourcing literature can be categorized into production and capacity planning [17,18], outsourcing contracts analysis [19,20] and suppliers selecting (such [21,22]. Among them, Kouvelis and Milner [23] present that demand volatility increases outsourcing rate while conversely supply variability decreases it for cases where supplier reacts to firm’s investments. Being slightly different and from the macro perspective, Steven et al. [22] analyze the linkage between supply chain sourcing strategies and quality recall. Xia et al. [24] evaluate the influence of globally outsourced inputs embedded in China’s exports on the well-known carbon outsourcing hypothesis. Bian et al. [25] examine the influences of different supply chain power structures on the strategies of service outsourcing.

Although the above literature analyzes carbon-emission reduction-relevant issues occurring in sourcing/outsourcing, it fails to incorporate the third-party contractor specializing in emission reduction into supply chain. Additionally, as far as we know, the existing literature almost ignores the role that the function outsourcing plays in carbon reduction. Hence, this is exactly what we do in this study to fill the gap. We find that the contractor’s investment coefficient heavily influences the supplier’s wholesale pricing decision when the supply chain member is demand risk-averse.

As stated in previous literature, we define a risk-averse firm as one unwilling to take a bet even when the bet is actuarially fair referring to some certainty position. In his pioneering work, Markowitz [26] firstly proposes the MV approach displaying elegance and simplicity to study financial risk management. Compared with expected utility models, such as von Neumann-Morgenstern utility and downside risk, the advantage of mean-variance (MV) and elegance lies in its intuitive explanation for diversity and simple computing procedures, whereas the principal critics are centered in its limitation of neglecting both the upside and downside deviations from the mean, which thus gives rise to the downside risk approach. However, Grootveld and Hallerbach [27] show that the downside risk approach and the variance approach did not really have a significant difference from the MV in many cases. Considering advantages and disadvantages of the MV analysis, we adopt it as the measure of utility in this study owing to its nature of applicability, intuitiveness and operability.

Some of the literature, such as [28,29,30], have developed their MV involved models. Chiu and Choi [28] address the coordination challenge for one risk-neutral manufacturer and multiple heterogeneous retailers. In a different scenario, Ray and Jenamani [29] employ a MV framework to handle disruption risk in a supply chain with one risk buyer and multiple suppliers. Focusing on risk-averse newsvendor models, Wu et al. [30] and Buzacott et al. [31] consider stock-out cost and commitment-option supply contracts other than mean variance objectives, respectively. Xia et al. [24] examine the impact of the retailers’ risk sensitivity on chain members’ optimal strategies by unveiling negative relationships between a retailer’s risk sensitivity and optimal service level as well as retail price. More information about supply chain risk analysis with mean-variance models can refer to Xiao and Yang [32] and Chiu and Choi [28].

Note that the aforementioned literature almost only compares the strategies between the risk-averse and risk-neutral scenarios by taking risk sensitivity as an exogenously given parameter. However, our study distinguishes from previous literature in many aspects. We regard the risk aversion within the supply chain as a controllable adjustment to confront demand volatility. Subsequently, we propose a contract to coordinate the risk-aversion embedded supply chain by maximizing the overall utility. Consequently, the present study potentially expands the research outreach depicted by the extant literature.

## 3. Preliminaries and Basic Models

We focus on a supply chain with one supplier (denoted *s*), one manufacturer (denoted *m*) and one contractor (denoted *c*). We denote *she* as the supplier and thus *he* as the corresponding representation for manufacturer. Suppose the supplier charges the manufacturer wholesale price w by providing him with materials with unit production cost $c_{s}$. The manufacturer provides consumers with low-carbon products charged at price p to cater their preference of low-carbon footprint with variable production cost $c_{m}$. The payment $F+\rho e+\theta e^{2}/2$ transferred from the manufacturer to the contractor is typically composed of three parts: *F* the fixed payment, ρe the revenue formed by carbon reduction level e times the payment level ρ and $\theta e^{2}/2$ the partial carbon-reduction cost undertaken by him. Considering the reality, the manufacturer’s undertaking cost would be less than the contractor’s total investment C(e), where we assume the cost coefficient θ is a constant parameter in the purpose of lowering the contracting complexity. For the sake of simplicity, we normalize *F* to zero, whereas it maintains the essence of the study.

We depict a linear demand function $D=\tilde{\alpha }-\beta p+\gamma e$ to capture consumer’s preference for low-carbon product, where $\tilde{\alpha }\equiv \alpha +\xi$, relates demand to retail price and carbon reduction level. Supposing that the random variable ξ follows the stochastic normal distribution $N(0,\sigma^{2})$, we rewrite the expected demand function as d=α+γe−βp, where α, γ and β are the potential demand, consumer’s sensitivity parameters in emission-reduction level and price, respectively. To avoid trivial issues, we assume $\alpha >\beta (c_{s}+c_{m})$.

Considering the diseconomies of carbon reduction, the fixed cost $C(e)\equiv ke^{2}/2$ we denote as emission-reducing cost will increase in carbon decrement with increasing marginal costs effect, namely, $c^{\prime }\left(e\right)>0$ and $c^{\prime \prime }\left(e\right)>0$. Related literature ([33,34,35]) generally also uses quadratic functions to describe the quantitative relationship between the environmental improvement and the corresponding expenditure.

Risk aversion may let the supplier or the manufacturer maximize their Mean-Variance-based objective function instead of traditional profit function. Moreover, we will omit the contractor’s risk tendency toward the demand fluctuation since the carbon reduction service conducted by the contractor is not affected by the demand volatility directly.

We use subscript ‘*m*’, ‘*s*’, ‘*c*’ and ‘*sc*’ to successively denote the manufacturer, the supplier, the contractor and the centralized supply chain, respectively. Note that the first superscript letter ‘*c*’ denotes the customizing case, and ‘*o*’ denotes the ordering case. Accordingly, the second superscript letter ‘*n*’ denotes the risk-neutral case, and ‘*a*’ denotes the risk-averse case. Table 1 summarizes the notations in this paper.

Hence, the problem of the contractor providing carbon emission service to the supply chain is to optimize the following objective function:

(1) $$\Pi_{c}=\rho e+\frac{1}{2}\theta e^{2}-\frac{1}{2}ke^{2}$$

where the first two terms are the revenue and the last term is carbon-reduction cost. Accordingly, the manufacturer’s problem is

(2) $$\Pi_{m}=(p-w-c_{m})\tilde{d}-\rho e-\theta e^{2}/2$$

where the first term is gross profit and the second term the service fee of carbon reduction paid to the contractor. The profit function of the supplier is given as follows.

(3) $$\Pi_{s}=(w-c_{s})D$$

where the right-hand is the net profit from the transaction with the manufacturer.

## 4. The Risk-Neutral Supplier and Manufacturer

In this subsection, we analyze the risk-neutral supply chain scenario where both the supplier and manufacturer are supposed risk-neutral as the benchmark for diverse scenarios. Here the decision variables are determined in the following order—the wholesale price, the emission-reduction price and reduction level, manufacturer’s setting retail price. During the game process, who and how to decide the emission-reduction price and carbon reduction level is vital for all chain members. When the manufacturer determines the emission-reduction price, he grasps the pricing power of the level of carbon reduction and could use it to guide the contractor’s decision of carbon reduction. However, in the other case where the manufacturer orders the carbon-reduction level while the contractor sets the emission-reduction price, the contractor may also utilize the pricing power to transfer his carbon-reduction cost to the manufacturer.

Therefore, to clarify the impact of different pricing power configurations on supply chain operations, we thus further devise the customizing and the ordering cases sequentially. In the former case, the manufacturer determines the emission-reduction price paid and then the contractor decides the carbon-reduction level. In the latter case, these two agents exchange their decision role just like that the contractor sells its product in a “take it or leave it” way and the manufacturer orders the quantity.

### 4.1. The Customizing Case

In this subsection we proceed to develop the associated Stackelberg game through backward induction according to the defined move sequence in the customizing case. We first analyze the contractor’s problem of determining carbon-reduction level given the manufacturer’s emission-reduction price and then the manufacturer’s decision regarding the emission-reduction price and retail price. Lastly, we discuss the supplier’s wholesale price problem.

As the contractor’s profit function may get the maximum value at a certain optimizer of carbon-reduction level since the second-order derivative condition $\partial^{2}\Pi_{c}/\partial e^{2}=-(k-\theta )<0$ holds, the first derivative of formula (1) below equal zero will characterize the optimal condition

(4) $$\frac{\partial \Pi_{c}}{\partial e}=\rho +\theta e-ke=0$$

Then we obtain the best response function of emission-reduction level as follows

(5) e=ρ/(k−θ)

which shows that the above response depends upon two parts—emission-reduction price, and the difference between the coefficient of carbon-reduction relevant fixed cost and the sharing level for the cost being apportioned. The first part of the emission-reduction price guides the contractor to determine the carbon-reduction level. If needing higher carbon reduction to attract more consumers, the manufacturer will promote the contractor through increasing emission-reduction price to enhance his carbon-reduction operations. The second part presents the difficulty of carbon reduction for the contractor, which will inversely affect the decision for the carbon-reduction level.

Substituting e in formula (5) into formula (2) transforms the manufacturer’s optimization problem as follows

(6) $$E(\Pi_{m})=(p-w-c_{m})\left(\alpha +\frac{\gamma \rho }{\left(k-\theta \right)}-\beta p\right)-\frac{\rho^{2}\left(2k-\theta \right)}{2{\left(k-\theta \right)}^{2}}$$

The second term of the right-hand side (RHS) of above function is the quadratic form of the cost paid for carbon reduction, which increases in the investment cost coefficient and indicates that the manufacturer gives the contractor a carbon-reduction capability-based payment. Note that although the contractor’s efficiency degree is positively correlated to the emission-reduction price offered by the manufacturer, this unnecessarily implies that the manufacturer prefers a low-efficiency contractor.

The Hessian matrix of the manufacturer’s objective function with respect to retail price and emission-reduction price is

(7) $$H_{m}\left(p,\rho \right)=\left[\begin{array}{cc} -2\beta & \gamma /(k-\theta )\\ \gamma /(k-\theta ) & -(2k+3\theta )/{\left(k-\theta \right)}^{2} \end{array}\right]$$

According to *Hurwitz theorem* ([33]), $E(\Pi_{m})$ is concave on (p,ρ) if and only if its Hessian matrix $H_{m}$ is negatively semi-definite, namely, $H_{m}\;{}^{’}s$ first leading principal minor −2β<0 and second leading principal minor determinant $2\beta (2k+3\theta )-\gamma^{2}\geq 0$ hold, respectively.

Letting the first partial derivative of $E(\Pi_{m})$ with respect to retail price equal zero as follows

(8) $$\frac{\partial E\left(\Pi_{m}\right)}{\partial p}=\alpha +\beta \left(w+c_{m}\right)+\frac{\gamma \rho }{k-\theta }-2\beta p=0$$

which characterizes below the best response function of p due to the aforesaid concave function $E(\Pi_{m})$

(9) $$p=\frac{\alpha +\beta \left(w+c_{m}\right)}{2\beta }+\frac{\gamma \rho }{2\beta (k-\theta )}$$

Note that the first term in the RHS of formula (9) reflects the influence of potential demand and wholesale price on retail price. The second term specifies the carbon reduction cost to be undertaken by customers. Furthermore, the second term can also be regarded as an increment of the potential demand from α to $\alpha +\frac{\gamma \rho }{k-\theta }$ due to the low-carbon preference of consumers.

Similarly, letting the first derivative $\frac{\partial E(\Pi_{m})}{\partial \rho }=\frac{\gamma }{k-\theta }\left(p-w-c_{m}\right)-\frac{2\rho }{k-\theta }-\frac{\theta \rho }{{\left(k-\theta \right)}^{2}}$ equal zero yields the best reactive function as follows

(10) $$\rho =\frac{\gamma (k-\theta )\left(p-w-c_{m}\right)}{2k-\theta }$$

Formula (10) indicates that the emission-reduction price will increase in terms of manufacturer’s profit margin as well as the low-carbon preference level. This payment mechanism runs as when the manufacturer shares his profit margin with the contractor and sets the sharing ratio according to the consumer’s low carbon preference. Furthermore, this formula also indicates that the contractor and the manufacturer have aligned interest regarding wholesale price and retail price by expecting higher retail price and lower wholesale price. Accordingly, combining formulas (9) and (10) gives us the following expressions

(11) $$p=\frac{(2k-\theta )\left[\alpha +\beta \left(w+c_{m}\right)\right]-\left(w+c_{m}\right)\gamma^{2}}{2\beta (2k-\theta )-\gamma^{2}}$$

(12) $$\rho =\frac{\gamma \left(k-\theta \right)\left(\alpha -\beta (w+c_{m})\right)}{2\beta (2k-\theta )-\gamma^{2}}$$

Now we turn to the supplier’s problem by deriving her expected profit function as follows

(13) $$E\left(\Pi_{s}\right)=\frac{\beta (2k-\theta )}{2\beta (2k-\theta )-\gamma^{2}}\left[\alpha -\beta \left(w+c_{m}\right)\right]\left(w-c_{s}\right)$$

Being easy to prove $E\left(\Pi_{s}\right)$ concave regarding the wholesale price, letting the first derivative $\frac{\partial E\left(\Pi_{s}\right)}{\partial w}=\frac{\beta (2k-\theta )}{2\beta (2k-\theta )-\gamma^{2}}\left(\alpha -2w\beta -\beta c_{m}+\beta c_{s}\right)$ equal zero yields the best response function

(14) $$w=\frac{\alpha }{2\beta }+\frac{c_{s}-c_{m}}{2}$$

Formula (14) shows that the wholesale price depends upon the potential demand, consumer’s price sensitivity level and unit production cost difference of supplier and manufacturer. However, consumers’ low-carbon preference level and investment coefficient will have little effect on the supplier’s decision as they neither assume carbon-reduction cost nor influence the third-party contractor’s decision.

Thus, substituting w from formulas (10)–(12) to formula (14) yields the following equilibriums

(15) $$p_{m}^{cn}=\frac{\alpha \left[3\beta \left(2k-\theta \right)-\gamma^{2}\right]+\beta \left[\beta \left(2k-\theta \right)-\gamma^{2}\right]\left(c_{m}+c_{s}\right)}{2\beta \left[2\beta \left(2k-\theta \right)-\gamma^{2}\right]}$$

(16) $$\rho_{m}^{cn}=\frac{\gamma \left(k-\theta \right)\left[\alpha -\beta \left(c_{m}+c_{s}\right)\right]}{2\left[2\beta \left(2k-\theta \right)-\gamma^{2}\right]}$$

(17) $$e_{ec}^{rn}=\frac{\gamma \left[\alpha -\beta \left(c_{m}+c_{s}\right)\right]}{2\left[2\beta \left(2k-\theta \right)-\gamma^{2}\right]}$$

The equilibrium profits of the supply chain members can be calculated as follows

(18) $$E\left(\Pi_{s}^{cn}\right)=\frac{\left(2k-\theta \right){\left[\alpha -\beta \left(c_{m}+c_{s}\right)\right]}^{2}}{4\left[2\beta \left(2k-\theta \right)-\gamma^{2}\right]}$$

(19) $$E\left(\Pi_{m}^{cn}\right)=\frac{\left(2k-\theta \right){\left[\alpha -\beta \left(c_{m}+c_{s}\right)\right]}^{2}}{8\left[2\beta \left(2k-\theta \right)-\gamma^{2}\right]}$$

(20) $$E\left(\Pi_{c}^{cn}\right)=\frac{\gamma^{2}\left(k-\theta \right){\left[\alpha -\beta \left(c_{m}+c_{s}\right)\right]}^{2}}{8{\left[2\beta \left(2k-\theta \right)-\gamma^{2}\right]}^{2}}$$

**Proposition** **1.** *According to the equilibriums in the customizing case, we can get the following relationships:* $\Pi_{c}^{*}=\frac{\gamma^{2}\left(k-\theta \right)\Pi_{m}^{*}}{2\left(2k-\theta \right)\left[2\beta \left(2k-\theta \right)-\gamma^{2}\right]}$ *or* $\Pi_{c}^{*}=\frac{\gamma^{2}\left(k-\theta \right)\left(\Pi_{c}^{*}+\Pi_{m}^{*}\right)}{6\left(2k-\theta \right)\left[2\beta \left(2k-\theta \right)-\gamma^{2}\right]}$*;* $\Pi_{m}^{*}=\frac{\Pi_{s}^{*}}{2}$ *or* $\Pi_{m}^{*}=\frac{1}{3}\left(\Pi_{m}^{*}+\Pi_{s}^{*}\right)$*.*

Observing the equilibriums can easily prove above proposition. Proposition 1 presents that the ratio of the contractor’s profit to the manufacturer’s is a constant. Furthermore, the relationship between the manufacturer and the contractor actually works the same as under a profit-sharing contract. That is, although they conduct separately, the chain system runs as if they construct a joint body. The contractor’s profit-sharing ratio increases in consumer preference level, but decreases in the price sensitivity and carbon-reduction investment coefficient. The same changing rule applies to the impact of parameters on emission-reduction price. The relationship between supplier’s and manufacturer’s profits can be stated as that of the contractor and the manufacturer, which we omit here for simplicity.

### 4.2. The Ordering Case

In this subsection, we first study the manufacturer’s problem on determining the retail price and the carbon-reduction level given the wholesale price and emission-reduction price. Afterwards, we analyze the supplier’s decision regarding the wholesale price and the contractor’s decision on the emission-reduction price. Note that here the sequence of analysis does not affect the results of our study since the manufacturer and the contractor move simultaneously.

Observing the below Hessian matrix of the manufacturer objective function in terms of the retail price and carbon reduction level

(21) $$H_{m}\left(p,e\right)=\left[\begin{array}{cc} -2\beta & \gamma \\ \gamma & -\theta \end{array}\right]$$

we can ensure the objective $E\left(\Pi_{m}\right)$ is concave on (p,e) if we assume $2\beta \theta -\gamma^{2}\geq 0$ to guarantee the Hessian matrix $H_{m}$ negative semi-definite.

Letting the first partial derivatives of $E\left(\Pi_{m}\right)$ with respect to the retail price and carbon reduction level equal zero to form an equation system

(22) $$\frac{\partial E\left(\Pi_{m}\right)}{\partial p}=\alpha -2\beta p+\gamma e+\beta w+\beta c_{m}=0$$

(23) $$\frac{\partial E\left(\Pi_{m}\right)}{\partial e}=\gamma \left(p-w-c_{m}\right)-\rho -e\theta =0$$

we solve this system to obtain following expressions

(24) $$p=\frac{\left(w+c_{m}\right)\left(\theta \beta -\gamma^{2}\right)+\theta \alpha -\gamma \rho }{2\beta \theta -\gamma^{2}}$$

(25) $$e=\frac{\gamma \left(\alpha -\beta \left(w+c_{m}\right)\right)-2\beta \rho }{2\beta \theta -\gamma^{2}}$$

Substituting formulas (24) and (25) into formula (1) can rewrite the contractor’s expected profit function as

(26) $$E\left(\Pi_{c}\right)=\frac{\left[2\beta \rho -\gamma \left(\alpha -\beta \left(w+c_{m}\right)\right)\right]\left[\gamma \left(k-\theta \right)\left(\alpha -\beta \left(w+c_{m}\right)\right)-2\rho \left(\beta \left(k+\theta \right)-\gamma^{2}\right)\right]}{2{\left(2\beta \theta -\gamma^{2}\right)}^{2}}$$

Subsequently, solving the equation generated by equaling below $E\left(\Pi_{ec}\right)$’s first partial derivative with respect to ρ zero

(27) $$\frac{\partial \Pi_{c}}{\partial \rho }=\frac{\gamma \left(2k\beta -\gamma^{2}\right)\left[\alpha -\beta \left(w+c_{m}\right)\right]-4\beta \rho \left[\beta \left(k+\theta \right)-\gamma^{2}\right]}{{\left(2\beta \theta -\gamma^{2}\right)}^{2}}$$

yields the equilibrium emission-reduction price

(28) $$\rho =\frac{\gamma \left(2k\beta -\gamma^{2}\right)\left[\alpha -\beta \left(w+c_{m}\right)\right]}{4\beta \left[\beta \left(k+\theta \right)-\gamma^{2}\right]}$$

Thereby we obtain the supplier’s expected profit function

(29) $$E\left(\Pi_{s}\right)=\frac{2\beta \left(k+\theta \right)-\gamma^{2}}{4\left[\beta \left(k+\theta \right)-\gamma^{2}\right]}\left[\alpha -\beta \left(w+c_{m}\right)\right]\left(w-c_{s}\right)$$

Deriving $E\left(\pi_{s}\right)$ with respect to w as follows

(30) $$\frac{\partial E\left(\Pi_{s}\right)}{\partial w}=\frac{\left(2k\beta -\gamma^{2})(\alpha -2w\beta -\beta c_{m}+\beta c_{s}\right)}{4k\beta -4\gamma^{2}}=0$$

we can obtain the equilibrium wholesale price

(31) $$w=\frac{\alpha }{2\beta }+\frac{c_{s}-c_{m}}{2}$$

**Remark** **1.** Comparing Equation (14) with Equation (31) demonstrates that the change of the contractor’s business model for carbon reduction does impact the supplier’s wholesale price a little. The logic behind this observation lies in the fact that we assume the carbon-reduction service is independent of unit level business regardless of the production volume.

Substituting Equation (31) into Equations (24), (25) and (28), we can obtain

(32) $$p=\frac{\alpha \left[6\beta \left(k+\theta \right)-5\gamma^{2}\right]+\beta \left[2\beta \left(k+\theta \right)-3\gamma^{2}\right]\left(c_{m}+c_{s}\right)}{8\beta \left[\beta \left(k+\theta \right)-\gamma^{2}\right]}$$

(33) $$e=\frac{\gamma \left[\alpha -\beta \left(c_{m}+c_{s}\right)\right]}{4\left[\beta \left(k+\theta \right)-\gamma^{2}\right]}$$

(34) $$\rho =\frac{\gamma (2k\beta -\gamma^{2})\left[\alpha -\beta (c_{m}+c_{s})\right]}{8\beta \left[\beta \left(k+\theta \right)-\gamma^{2}\right]}$$

Correspondingly, the equilibrium profits of chain members are expressed as follows

(35) $$E\left(\pi_{s}\right)=\frac{\left[2\beta \left(k+\theta \right)-\gamma^{2}\right]{\left[\alpha -\beta \left(c_{m}+c_{s}\right)\right]}^{2}}{16\beta \left[\beta \left(k+\theta \right)-\gamma^{2}\right]}$$

(36) $$E\left(\pi_{m}\right)=\frac{\left[4k^{2}\beta^{2}+3\gamma^{4}-6\beta \gamma^{2}\theta +4\beta^{2}\theta^{2}+8k\beta \left(-\gamma^{2}+\beta \theta \right)\right]{\left[\alpha -\beta \left(c_{m}+c_{s}\right)\right]}^{2}}{64\beta {\left[\gamma^{2}-\beta \left(k+\theta \right)\right]}^{2}}$$

(37) $$E\left(\pi_{c}\right)=\frac{\gamma^{2}{\left[\alpha -\beta \left(c_{m}+c_{s}\right)\right]}^{2}}{32\beta \left[\beta \left(k+\theta \right)-\gamma^{2}\right]}$$

**Proposition** **2.** *Comparison between the customizing case and the order one shows that the emission-reduction price in the latter case is higher than the former one, whereas we have* $p_{m}^{on}\geq p_{m}^{cn}$ *and* $e_{m}^{on}\geq e_{c}^{cn}$ *when* $k\geq 2\theta -\gamma^{2}/2\beta$*, or* $p_{m}^{on}<p_{m}^{cn}$ *and* $e_{m}^{on}<e_{c}^{cn}$ *when* $k<2\theta -\gamma^{2}/2\beta$*.*

This proposition indicates that the customizing case, compared with the order case, possesses higher retail price, lower carbon-reduction level and lower emission-reduction price. We may explain this phenomenon in the following aspects. In the ordering case, the contractor retains the right of pricing emission-reduction but leaves the right of determining carbon-reduction level to the manufacturer, which, hence, will incentivize the contractor to set a high price to raise his profit margin. However, in the customizing case, the manufacturer is prone to compress the emission-reduction level herein ordered by him for lowering the cost undertaken. To sum up, both high carbon-reduction level and emission-reduction price push up the average unit cost, which subsequently raises the retail price in the ordering case since the manufacturer transfers extra cost to the consumer.

**Proposition** **3.** *Compared with the customizing case, the ordering case holds better performance for the supply chain, higher profit for the contractor, lower profit for the manufacturer and a higher demand. Moreover, supplier’s profit in the order case is higher than that in the customizing case when* $k>2\theta -\gamma^{2}/2\beta$*.*

The proposition is easy to prove and indicate that the change of carbon-reduction model of the contractor may profoundly impact supply chain performances, which can be reflected in the fact that the order case presents Pareto improvement for the manufacturer and contractor compared with other cases. However, the supplier has a higher profit in the customizing case only when the contractor holds carbon-reduction efficiency.

Intuitively, obtaining the pricing power of carbon reduction could give a chain member the initiative priority in transactions. However, the above result shows that the contractor owning the pricing power in the ordering case performs better than in the other case. Simultaneously, we can derive from the customizing case manufacturer’s irrationality on carbon reduction that pricing tends to cut the payment level to control his cost in carbon reduction, which thus makes the role of carbon reduction in promoting demand lack attention. Additionally, the contractor determines its carbon-reduction in light of the manufacturer’s payment level, which creates a vicious circle and curbs the improvement of bilateral profits, whereas the ordering case precludes this issue. Therefore, the manufacturer and contractor would prefer the ordering case considering that the supplier, scarcely affects the ownership of pricing power on carbon reduction. Compared with the customizing case, the profits of the supply system and the manufacturer get better in the ordering case, while the contractor’s profit gets worse.

Conducting the numerical analysis, we intend to show how the pricing modes and the coefficients of carbon-reduction investment and sharing cost impact the supply chain performance. The parameters are set as follows, α=200, β=2, γ=40, $c_{s}=15$, $c_{m}=12$, k=1500. $\alpha =200,\;\beta =2,\;\gamma =40,\;c_{s}=15,\;c_{m}=12,\;k=1500$. We select the evaluation for parameters based on the previous analytical mathematic relationship between themselves.

**Figure 1:**
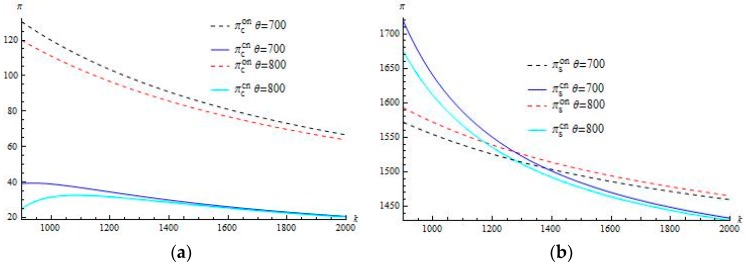

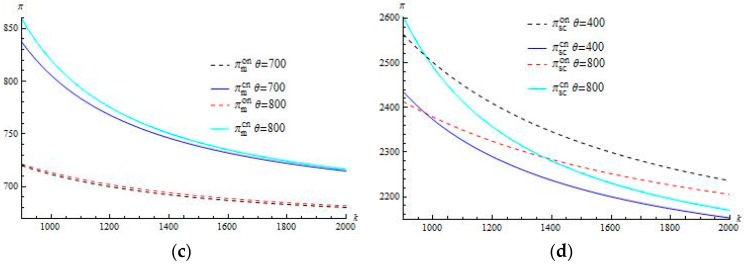
(**a**) Comparing $\pi_{c}^{cn}$ and $\pi_{c}^{on}$ as *k* varies; (**b**) Comparing $\pi_{s}^{cn}$ and $\pi_{s}^{on}$ as *k* varies; (**c**) Comparing $\pi_{m}^{cn}$ and $\pi_{m}^{on}$ as *k* varies; (**d**) Comparing $\pi_{ec}^{cn}$ and $\pi_{ec}^{on}$ as *k* varies.

Generally, for the manufacturer the investment cost shared means the burden ruining his profit, whereas it belongs to the contractor’s revenue. However, the numerical analysis gives a very different result. Figure 1a demonstrates that in the customizing case the contractor’s profit decreases in the investment-sharing coefficient θ while all the profits of the supplier, the manufacturer and supply chain increase in θ. However, in the ordering case all the profits of the contractor, the supplier and supply chain decrease in θ, but the manufacturer’s profit decreases in θ.

### 4.3. The Centralized Risk-Averse Supply Chain

As done traditionally, we study in this subsection the centralized risk-averse supply chain as the benchmark for analyzing the subsequent decentralized one. The centralized supply chain here holds the same chain structure including the supplier, the manufacturer and the contractor, which works when there exists a dictator integrating all decision-making rights. Suppose the chain dictator in the centralized system is risk averse and conservative about demand risk assessing the total utility via the following MV function U=E(Π)−λσ, where the first term of RHS is the mean profit, and the second the risk cost. Note that here the parameter λ reflects the dictator’s attitude towards the demand uncertainty and σ the standard deviation of its random profit. The aforesaid utility function implies that the dictator controls his risk through the parameter λ to adjust the difference between the mean and the variance of its random profit.

We denote $\lambda_{cs}$ as the risk-aversion level of the centralized supply chain, which reflects the dictator’s dominant concerns on the demand volatility. The expected supply chain profit and its variance are shown as follows, respectively

(38) $$E\left(\Pi_{sc}\right)=\left(p-c_{s}-c_{m}\right)\left(\alpha +\gamma e-\beta p\right)-\frac{1}{2}ke^{2}$$

(39) $$Var_{sc}=E\left\{{\left[\Pi_{cs}-E\left(\Pi_{cs}\right)\right]}^{2}\right\}={\left(p-c_{s}-c_{m}\right)}^{2}\sigma^{2}$$

Thus, the supply chain utility can be expressed as below

(40) $$U_{sc}=E\left(\Pi_{sc}\right)-\lambda \sqrt{Var_{sc}}=\left(p-c_{s}-c_{m}\right)\left(d-\lambda_{sc}\sigma \right)-\frac{1}{2}ke^{2}$$

We can explain $d-\lambda_{m}\sigma$ as an adjustment to the mean from which measures the dispersion. Observing the above functions, we know the probability that the demand $E\left(d\right)-\lambda_{m}\sigma$ will be satisfied increases in the risk-aversion level. For example, when choosing $\lambda_{m}=1$ and $\lambda_{m}=2$, the probabilities satisfying the demand are 84.1% and 97.7%, respectively. Thus, we can obtain the following remark.

**Remark** **2.** The risk-aversion level functions as an adjustment to the mean (average) demand.

**Proposition** **4.** *By assuming* $2\beta k-\gamma^{2}>0$ *to ensure the results positive, the optimal decision variables of the centralized risk-averse supply chain are as follows. (Proof please see the Appendix A)*

(41) $$p_{sc}^{cs}=\frac{k\left(\alpha -\lambda_{cs}\sigma \right)+\left(k\beta -\gamma^{2}\right)\left(c_{m}+c_{s}\right)}{2k\beta -\gamma^{2}}$$

(42) $$e_{ec}^{cs}=\frac{\gamma \left[\alpha -\beta \left(c_{m}+c_{s}\right)-\lambda_{cs}\sigma \right]}{2k\beta -\gamma^{2}}$$

## 5. The Risk-Averse Supplier and Manufacturer

### 5.1. The Customizing Case with Risk-Aversion

In this section, we concentrate on the decentralized supply chain having the supplier and the manufacturer both risk-averse. Thus, they measure their utilities via the MV method to reflect relevant risks in their objective functions, respectively. In addition, we omit the sole risk-averse supplier ($\lambda_{s}>0$ and $\lambda_{m}=0$) or manufacturer ($\lambda_{s}=0$ and $\lambda_{m}>0$) cases since they are just special cases embraced in our model here. The variances of the supplier’s and the manufacturer’s profits are given as follows, respectively

(43) $$Var\left(\Pi_{s}\right)=E\left\{{\left[\Pi_{s}-E\left(\Pi_{s}\right)\right]}^{2}\right\}={\left(w-c_{s}\right)}^{2}\sigma^{2}$$

(44) $$Var\left(\Pi_{m}\right)=E\left\{{\left[\Pi_{m}-E\left(\Pi_{m}\right)\right]}^{2}\right\}={\left(p-w-c_{m}\right)}^{2}\sigma^{2}$$

As the move sequence in the decentralized risk-averse supply chain resembles that of the decentralized risk-neutral system, the resulted analysis on the contractor’s problem is almost the same as the preceding risk-neutral case. So now we turn to the manufacturer’s problem as follows

(45) $$\underset{p,\rho }{\max}\;U_{m}=\left(p-w-c_{m}\right)\left(\alpha -\beta p+\frac{\gamma \rho }{k-\theta }\right)-\lambda_{m}\left(p-w-c_{m}\right)\sigma -\frac{\rho^{2}\left(2k-\theta \right)}{2{\left(k-\theta \right)}^{2}}$$

Formula (45) indicates that manufacturer’s risk aversion diminishes his own utility compared with that of the risk-neutral case. Since functions (7) and (45) have exactly the same Hessian matrix with respect to p and ρ, the condition for ensuring the existence of the optimal utility in the risk-averse case is also the same as that in the risk-neutral one.

Letting below the first partial derivative of (45) with respect to retail price p equal zero characterizes the optimal condition of retail pricing

(46) $$\frac{\partial E\left(\Pi_{m}\right)}{\partial p}=\alpha +\beta \left(w+c_{m}\right)+\frac{\gamma p}{k-\theta }-2\beta p-\lambda_{m}\sigma =0$$

Thus, we obtain the best response function of p as follows

(47) $$p=\frac{\alpha +\beta \left(w+c_{m}\right)}{2\beta }+\frac{\gamma \rho }{2\beta \left(k+\theta \right)}-\frac{\lambda_{m}\sigma }{2\beta }$$

**Remark** **3.** The manufacturer’s risk-aversion cuts down his retail price.

Compared with the risk-neutral case, the retail price in the risk-averse is lower due to the demand fluctuation and risk attitude. The reason why this case happens can be explained similarly to how the risk-averse decision-maker intends to attract the price-sensitive consumers by reducing retail price to evade the demand risk. Although the manufacturer confronts the same demand fluctuation with the supplier, their risk-aversion levels are different.

Combining the first partial derivative of formula (45) regarding the emission-reduction price and expression (47) yields the following results

(48) $$\rho =\frac{\gamma \left(k-\theta \right)\left[\alpha -\beta \left(w+c_{m}\right)-\sigma \lambda_{m}\right]}{2\beta \left(2k-\theta \right)-\gamma^{2}}$$

(49) $$p=\frac{(2k-\theta )\left[\alpha +\beta \left(w+c_{m}\right)-\sigma \lambda_{m}\right]-\left(w+c_{m}\right)\gamma^{2}}{2\beta \left(2k-\theta \right)-\gamma^{2}}$$

The supplier’s utility function can be expressed as follows

(50) $$U_{s}=\left(w-c_{s}\right)d-\lambda_{s}\left(w-c_{s}\right)\sigma$$

After substituting formulas (5), (48) and (49) into (50), letting the following first derivative of supplier’s utility function with respect to the wholesale price equal zero characterizes the optimal condition.

(51) $$\frac{\partial U_{s}}{\partial w}=\frac{\beta \left(\alpha -2w\beta +\beta \left(c_{s}-c_{m}\right)\right)\left(2k-\theta \right)+\left(2k\beta -\gamma^{2}-\beta \theta \right)\sigma \lambda_{m}+\left(-4k\beta +\gamma^{2}+2\beta \theta \right)\sigma \lambda_{s}}{4k\beta -\gamma^{2}-2\beta \theta }=0$$

Accordingly, we obtain the best response function of wholesale price as follows

(52) $$w_{m}^{ds}=\frac{\alpha }{2\beta }+\frac{c_{s}-c_{m}}{2}+\frac{\sigma \left[\left(\beta \left(2k-\theta \right)-\gamma^{2}\right)\lambda_{m}-\left(2\beta \left(2k-\theta \right)-\gamma^{2}\right)\lambda_{s}\right]}{2\beta^{2}\left(2k-\theta \right)}$$

The third term in the RHS of formula (52) presents the joint impact of supplier’s and manufacturer’s risk attitude on the supplier’s wholesale-pricing decision. From formula (52), the supplier will utilize the manufacturer’s risk aversion to raise her wholesale price, whereas her own risk aversion lets it drop off. Moreover, as shown in (52) the weight coefficient associated with the manufacturer’s risk aversion is less than the supplier’s, which unnecessarily implies the supplier’s risk aversion impact is greater than that of the manufacturer.

**Remark** **4.** *The wholesale price decreases in the standard deviation of market demand if and only if* $k<\frac{\gamma^{2}}{2\beta }+\frac{\theta }{2}$*.*

The demand deviation definitely holds the negative impact on the retail price, whereas the wholesale price increases with increasing demand deviation when the risk-aversion impact of the manufacturer is greater than that of the supplier.

**Remark** **5.** *As the supplier extends her concern range when she is risk-averse, her equilibrium wholesale price decreases in carbon-reduction coefficient when* $\lambda_{s}>\lambda_{m}$ *. Otherwise, it increases.*

Formula (52) also shows that the contractor’s emission-reduction investment coefficient in the risk-averse case also affects the supplier’s wholesale price decision, which indicates that either the supplier’s or the manufacturer’s risk aversion may generate and even extend the supplier’s concerning range.

How the carbon-reduction investment coefficient impacts on the wholesale price heavily depends upon the resulting comparison of supplier’s and manufacturer’s risk aversions. When the manufacturer has higher risk aversion than the supplier’s, the wholesale price will increase in the carbon-reduction coefficient because the supplier can charge high wholesale prices to increase her utility by exploiting the manufacturer’s risk aversion. In the opposite situation, the corresponding explanation is that the supplier may reduce her wholesale price to relieve the pressure from the contractor’s inefficiency to attract the price-sensitive consumers.

And then we have the equilibrium solutions of all decision variables as follows

(53) $$p_{m}^{cn}=\frac{\alpha \left[3\beta \left(2k-\theta \right)-\gamma^{2}\right]}{2\beta \left[2\beta \left(2k-\theta \right)-\gamma^{2}\right]}+\frac{\left[\beta \left(2k-\theta \right)-\gamma^{2}\right]\left(c_{m}+c_{s}\right)}{2\left[2\beta \left(2k-\theta \right)-\gamma^{2}\right]}+\frac{\left[2\gamma^{4}-{\left(\beta \left(2k-\theta \right)-\gamma^{2}\right)}^{2}\right]\sigma \lambda_{m}}{2\beta^{2}\left(2k-\theta \right)\left(4k\beta -\gamma^{2}-2\beta \theta \right)}+\frac{\left[\beta \left(2k-\theta \right)-\gamma^{2}\right]\sigma \lambda_{s}}{2\beta^{2}\left(2k-\theta \right)}$$

(54) $$\rho_{m}^{cn}=\frac{\gamma \left(k-\theta \right)\left(\alpha -\beta \left(c_{m}+c_{s}\right)\right)}{2\left(2\beta \left(2k-\theta \right)-\gamma^{2}\right)}+\frac{\gamma \left(k-\theta \right)\left(\left(2\beta \left(2k-\theta \right)-\gamma^{2}\right)\sigma \lambda_{s}-\left(3\beta \left(2k-\theta \right)-\gamma^{2}\right)\sigma \lambda_{m}\right)}{2\beta \left(2k-\theta \right)\left(2\beta \left(2k-\theta \right)-\gamma^{2}\right)}$$

(55) $$e_{c}^{cn}=\frac{\gamma \left(\alpha -\beta \left(c_{m}+c_{s}\right)\right)}{2\left(2\beta \left(2k-\theta \right)-\gamma^{2}\right)}+\frac{\gamma \left(\left(2\beta \left(2k-\theta \right)-\gamma^{2}\right)\sigma \lambda_{s}-\left(3\beta \left(2k-\theta \right)-\gamma^{2}\right)\sigma \lambda_{m}\right)}{2\beta \left(2k-\theta \right)\left(2\beta \left(2k-\theta \right)-\gamma^{2}\right)}$$

The equilibrium utilities of all chain members are

(56) $$U_{c}^{cn}=\frac{\gamma^{2}\left(k-\theta \right){\left(\beta \left(2k-\theta \right)\left(\alpha -\beta \left(c_{m}+c_{s}\right)\right)-\left(3\beta \left(2k-\theta \right)-\gamma^{2}\right)\sigma \lambda_{m}+\left(2\beta \left(2k-\theta \right)-\gamma^{2}\right)\sigma \lambda_{s}\right)}^{2}}{8\beta^{2}{\left(2k-\theta \right)}^{2}{\left(2\beta \left(2k-\theta \right)-\gamma^{2}\right)}^{2}}$$

(57) $$U_{m}^{cn}=\frac{{\left(\beta \left(2k-\theta \right)\left(\alpha -\beta \left(c_{m}+c_{s}\right)\right)-\left(3\beta \left(2k-\theta \right)-\gamma^{2}\right)\sigma \lambda_{m}+\left(2\beta \left(2k-\theta \right)-\gamma^{2}\right)\sigma \lambda_{s}\right)}^{2}}{8\beta^{2}\left(2k-\theta \right)\left(2\beta \left(2k-\theta \right)-\gamma^{2}\right)}$$

(58) $$U_{s}^{cn}=\frac{{\left(\beta \left(2k-\theta \right)\left(\alpha -\beta \left(c_{m}+c_{s}\right)\right)+\left(\beta \left(2k-\theta \right)-\gamma^{2}\right)\sigma \lambda_{m}-\left(2\beta \left(2k-\theta \right)-\gamma^{2}\right)\sigma \lambda_{s}\right)}^{2}}{4\beta^{2}\left(2k-\theta \right)\left(2\beta \left(2k-\theta \right)-\gamma^{2}\right)}$$

**Remark** **6.** *The risk aversion can cause the change on the ratio of supplier’s utility to the manufacturer’s when* $\lambda_{s}\neq \lambda_{m}$*, whereas the ratio of the manufacturer’s utility to the contractor’s does not matter.*

The above conclusion can be derived from the formulas (56) and (57), namely, $U_{c}=\frac{\gamma^{2}\;U_{m}\left(k-\theta \right)}{\left(2k-\theta \right)\left(2\beta \left(2k-\theta \right)-\gamma^{2}\right)}$. This phenomenon occurs since the contractor is risk-neutral in terms of demand risk, yet the risk-aversion changes the utility relationship between the manufacturer and supplier unless they have equal risk-aversion levels ($\lambda_{m}=\lambda_{s}$).

**Remark** **7.** *Both the manufacturer’s and the contractor’s utilities decrease in* $\lambda_{m}$ *and in* $\lambda_{s}$ *if* $\frac{\gamma^{2}}{2\beta }+\frac{\theta }{3}<k<\frac{\gamma^{2}}{2\beta }+\frac{\theta }{2}$*, whereas then the supplier’s utility increases both in* $\lambda_{m}$ *and in* $\lambda_{s}$*. The manufacturer’s and the contractor’s utilities decrease in* $\lambda_{m}$ *but increase in* $\lambda_{s}$ *if* $k>\frac{\gamma^{2}}{2\beta }+\frac{\theta }{2}$*. Therefore, the manufacturer may accept the demand risk in a certain level to maximize his utility. Like the case for the manufacturer, the supplier also suffers from her own risk-averse level.*

### 5.2. The Ordering Case with Risk-Aversion

As for the ordering case, the related properties of the decision variables resemble that of Section 4.2 and Section 5.1 due to the similar relevant decision sequence. We present the results but omit the deriving process.

(59) $$w_{s}^{do}=\frac{\alpha +\beta \left(c_{s}-c_{m}\right)}{2\beta }+\frac{\left(2\beta \left(k+\theta \right)-\gamma^{2}\right)\sigma \lambda_{m}-4\left(\beta \left(k+\theta \right)-\gamma^{2}\right)\sigma \lambda_{s}}{2\beta \left(2\beta \left(k+\theta \right)-\gamma^{2}\right)}$$

(60) $$\begin{array}{ll} p_{m}^{do} & =\frac{\alpha \left(6\beta \left(k+\theta \right)-5\gamma^{2}\right)+\beta \left(2\beta \left(k+\theta \right)-3\gamma^{2}\right)\left(c_{m}+c_{s}\right)}{8\beta \left(k\left(k+\theta \right)-\gamma^{2}\right)}\\ & +\frac{\left({\left(2\beta \left(k+\theta \right)+\gamma^{2}\right)}^{2}-8\gamma^{4}\right)\sigma \lambda_{m}-4\left(2\beta \left(k+\theta \right)-3\gamma^{2}\right)\left(\beta \left(k+\theta \right)-\gamma^{2}\right)\sigma \lambda_{s}}{8\beta \left(\beta \left(k+\theta \right)-\gamma^{2}\right)\left(2\beta \left(k+\theta \right)-\gamma \right)} \end{array}$$

(61) $$e_{m}^{do}=\frac{\gamma \left(\alpha -\beta \left(c_{m}+c_{s}\right)\right)}{4\left(k\beta -\gamma^{2}\right)}+\frac{\gamma \left(4\left(\beta \left(k+\theta \right)-\gamma^{2}\right)\sigma \lambda_{s}-\left(6\beta \left(k+\theta \right)-5\gamma^{2}\right)\sigma \lambda_{m}\right)}{4\left(2\beta \left(k+\theta \right)-\gamma^{2}\right)\left(\beta \left(k+\theta \right)-\gamma^{2}\right)}$$

(62) $$\begin{array}{l} \rho_{ec}^{do}=\frac{\gamma \left(2k\beta -\gamma^{2}\right)\left(\alpha -\beta \left(c_{m}+c_{s}\right)\right)}{8\beta \left(\beta \left(k+\theta \right)-\gamma^{2}\right)}\\ +\frac{\gamma \left(2k\beta -\gamma^{2}\right)\left(4\left(\beta \left(k+\theta \right)-\gamma^{2}\right)\sigma \lambda_{s}-\left(6\beta \left(k+\theta \right)-5\gamma^{2}\right)\sigma \lambda_{m}\right)}{8\beta \left(\beta \left(k+\theta \right)-\gamma^{2}\right)\left(2\beta \left(k+\theta \right)-\gamma^{2}\right)} \end{array}$$

(63) $$U_{ec}^{do}=\frac{\gamma^{2}{\left(\left(2\beta \left(k+\theta \right)-\gamma^{2}\right)\left(\alpha -\beta c_{m}-\beta c_{s}\right)-\left(6\beta \left(k+\theta \right)-5\gamma^{2}\right)\sigma \lambda_{m}+4\left(\beta \left(k+\theta \right)-\gamma^{2}\right)\sigma \lambda_{s}\right)}^{2}}{32\beta {\left(2\beta \left(k+\theta \right)-\gamma^{2}\right)}^{2}\left(\beta \left(k+\theta \right)-\gamma^{2}\right)}$$

(64) $$U_{s}^{do}=\frac{{\left[\left(2\beta \left(k+\theta \right)-\gamma^{2}\right)\left(\alpha -\beta c_{m}-\beta c_{s}\right)+\left(2\beta \left(k+\theta \right)-3\gamma^{2}\right)\sigma \lambda_{m}-4\left(\beta \left(k+\theta \right)-\gamma^{2}\right)\sigma \lambda_{s}\right]}^{2}}{16\beta \left(\beta \left(k+\theta \right)-\gamma^{2}\right)\left(2\beta \left(k+\theta \right)-\gamma^{2}\right)}$$

(65) $$U_{m}^{do}=\frac{\left[{\left(2\beta \left(k+\theta \right)-\gamma^{2}\right)}^{2}+2\gamma^{2}\left(\gamma^{2}+2\beta \theta \right)\right]}{64\beta {\left(2\beta \left(k+\theta \right)-\gamma^{2}\right)}^{2}{\left(\beta \left(k+\theta \right)-\gamma^{2}\right)}^{2}}{\left[\left(2\beta \left(k+\theta \right)-\gamma^{2}\right)\left(\alpha -\beta c_{m}-\beta c_{s}\right)-\left(6\beta \left(k+\theta \right)-5\gamma^{2}\right)\sigma \lambda_{m}+4\left(\beta \left(k+\theta \right)-\gamma^{2}\right)\sigma \lambda_{s}\right]}^{2}$$

## 6. Numerical Analyses and Managerial Insights

Considering the complexity of all chain members’ utility form, we conduct numerical analysis here to investigate the effects of risk aversion and demand volatility on supply chain performance and reveal some managerial insights that can be obtained from the preceding analysis. Set the parameters as follows: α=200, β=2, γ=40, $c_{s}=15$, $c_{m}=12$, k=1500.

**Figure 2:**
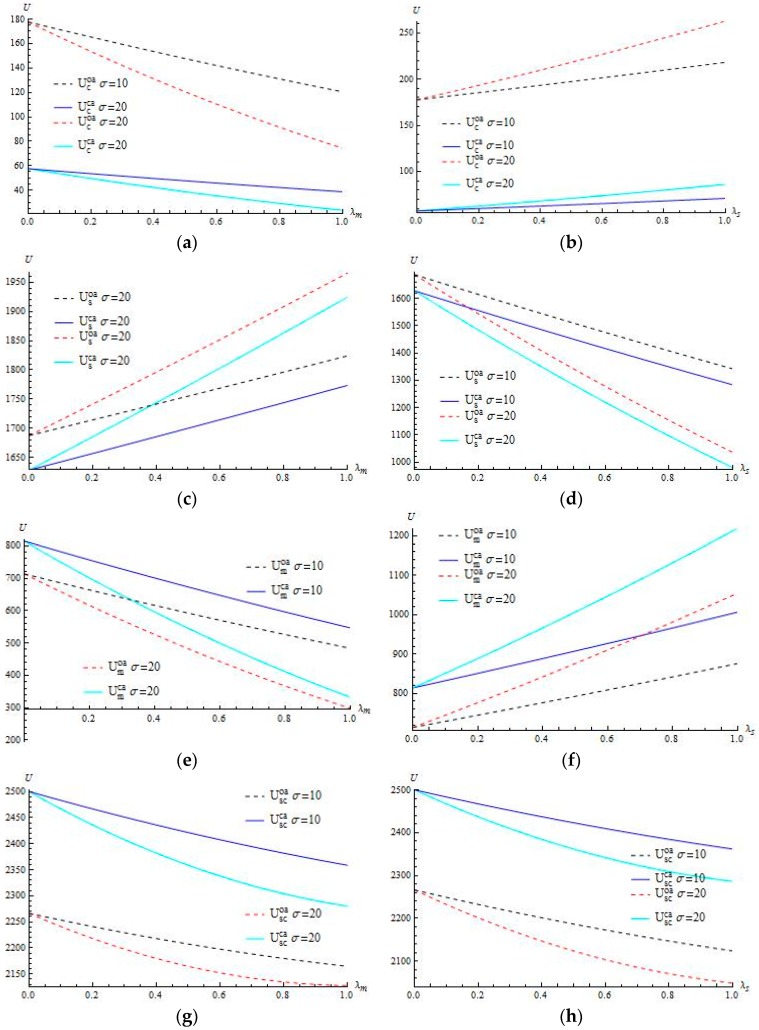
The impact of risk-aversion levels on utilities of chain members. (**a**) Comparing $U_{c}^{oa}$ and $U_{c}^{ca}$ as $\lambda_{m}$ varies when $\lambda_{s}=0$; (**b**) Comparing $U_{c}^{oa}$ and $U_{c}^{ca}$ as $\lambda_{s}$ varies when $\lambda_{m}=0$; (**c**) Comparing $U_{s}^{oa}$ and $U_{s}^{ca}$ as $\lambda_{m}$ varies when $\lambda_{s}=0$; (**d**) Comparing $U_{s}^{oa}$ and $U_{s}^{ca}$ as $\lambda_{s}$ varies when $\lambda_{m}=0$; (**e**) Comparing $U_{m}^{oa}$ and $U_{m}^{ca}$ as $\lambda_{m}$ varies when $\lambda_{s}=0$; (**f**) Comparing $U_{m}^{oa}$ and $U_{m}^{ca}$ as $\lambda_{s}$ varies when $\lambda_{m}=0$; (**g**) Comparing $U_{sc}^{oa}$ and $U_{sc}^{ca}$ as $\lambda_{m}$ varies when $\lambda_{s}=0$; (**h**) Comparing $U_{sc}^{oa}$ and $U_{sc}^{ca}$ as $\lambda_{s}$ varies when $\lambda_{m}=0$.

Figure 2a, b show that the contractor’s utility decreases in the manufacturer’s risk-aversion level $\lambda_{m}$ but increases in supplier’s $\lambda_{s}$. Moreover, Figure 2c,d demonstrates that the supplier’s utility increases in $\lambda_{m}$ when there is an increase in $\lambda_{s}$. The utility of each agent decreases in its own risk-aversion level while increases in its partner’s (shown in Figure 2a,b) in the interval $\lambda_{m},\;\lambda_{s}\in \left(0,1\right)$. This indicates that the firm may benefit from risk aversions of its trading partners. Therefore, it is probably effective from the perspective of supply chain operations to transact with a risk-averse partner to improve its utility, whereas as to its own risk attitude, holding low risk-aversion and tolerating proper risk of demand volatility can be beneficial to its utility.

The contractor’s utility in the ordering case is higher than that in the customizing case with the utility difference incurred by these two modes decreasing in $\lambda_{m}$. Moreover, the numerical study in Figure 2 also reveals that the contractor’s utility decreases in demand deviation and the difference aroused by two different deviations increases in $\lambda_{m}$.

Additionally, the chain utility decreasing both in risk-aversion level and demand dispersing extent is intuitive. Although the risk aversion on demand volatility results in conservative behaviors, reasonably, appropriate risk-aversion level contributes to stabilize operations strategies and tactics. Conversely, excessively pursuing stable operations usually leads to the loss of important opportunities. Therefore, in order to maximize the effectiveness of the supply chain, decision-makers should be appropriate to reduce the degree of risk aversion to improve its utility. The impact of the demand deviation in supply chain utility is also crucial. Apparently, reducing demand deviation helps to lift supply chain utility. Hence, the chain members should be dedicated to applying the modern information technology methods to collect market demand information while necessarily reducing the possible bullwhip effect.

## 7. Coordination Contract for Decentralized Risk-Averse Supply Chain

Upon understanding the sense of the risk-aversion level, we design some contract to coordinate the supply chain through adjusting the risk-aversion levels of the supplier and manufacturer to let the retail price and the carbon reduction level in the decentralized supply chain equal to those in the centralized supply chain.

**Proposition** **5.** *In the customizing case, the supply chain is coordinated when parameters* $\lambda_{s}$ *and* $\lambda_{m}$ *hold the following assigned values*

(66) $$\begin{array}{l} \lambda_{s}=\frac{1}{\left(2k\beta -\gamma^{2}\right)\left(4k\beta -\gamma^{2}-2\beta \theta \right)\sigma }[\beta \left(6k^{2}\beta +\beta \theta^{2}-k\left(\gamma^{2}+5\beta \theta \right)\right)\left(\alpha -\beta \left(c_{m}+c_{s}\right)\right)\\ +\left(2k^{2}\beta^{2}+\gamma^{4}+2\beta \gamma^{2}\theta -\beta^{2}\theta^{2}+k\beta \left(-5\gamma^{2}+\beta \theta \right)\right)\sigma \lambda_{sc}] \end{array}$$

(67) $$\lambda_{m}=\frac{\beta \left(k-\theta \right)\left(\beta \left(c_{m}+c_{s}\right)\alpha \right)+\left(3k\beta -\gamma^{2}-\beta \theta \right)\sigma \lambda_{sc}}{\left(2k\beta -\gamma^{2}\right)\sigma }$$

In the ordering case, the supply chain is coordinated when the following parameters evaluation hold

(68) $$\begin{array}{l} \lambda_{s}=\frac{1}{8\left(2k\beta -\gamma^{2}\right)\left(\beta \left(k+\theta \right)-\gamma^{2}\right)\sigma }[\left(8k^{2}\beta^{2}+{\left(\gamma^{2}-2\beta \theta \right)}^{2}+2k\beta \left(-5\gamma^{2}+6\beta \theta \right)\right)\left(\alpha -\beta \left(c_{m}+c_{s}\right)\right)\\ +\left(8k^{2}\beta^{2}+7\gamma^{4}-4\beta \gamma^{2}\theta -4\beta^{2}\theta^{2}+2k\beta \left(-7\gamma^{2}+2\beta \theta \right)\right)\sigma \lambda_{sc}] \end{array}$$

(69) $$\lambda_{m}=\frac{\left(\gamma^{2}-2\beta \theta \right)\left(\alpha -\beta \left(c_{m}+c_{s}\right)\right)+\left(2\beta \left(2k+\theta \right)-3\gamma^{2}\right)\sigma \lambda_{sc}}{2\left(k\beta -\gamma^{2}\right)\sigma }$$

**Proof.**  To achieve supply chain coordination, we adjust the level of risk aversion and let the following equations hold.

(70) $$p_{m}^{cs}=p_{m}^{ds}$$

(71) $$e_{ec}^{cs}=e_{ec}^{ds}$$

From the above equations, we derive the corresponding solutions of the risk-averse level of the supplier and manufacturer, respectively. As shown in formula (67), inequality $\alpha >\beta (c_{m}+c_{s})+3-\frac{\gamma^{2}}{k\beta }$ implies $\lambda_{m}<0$. That is, in order to coordinate supply chain, the manufacturer should be risk appetite.

Note that, to the best of our knowledge, the existing literature seldom tries to optimize the agent’s risk-aversion level for coordinating supply chains. One may argue that the risk-aversion level should be treated as an exogenous variable or a certain parameter of the system since risk-aversion is one kind of stable psychological status of the decision maker. However, the risk-aversion level in current study decision process with the MV model works as an adjustment to the expected demand based on the decision maker’s knowledge of the demand volatility as we note previously. When determining the risk-aversion level, the decision maker may turn to some experts for suggestions. Therefore, taking the risk-aversion level as an indigenous variable is acceptable academically and practically.

## 8. Conclusions

This paper studies the risk aversion management problem in low-carbon supply chains in the presence of emission abatement outsourcing. We analyze a supply chain consisting of one supplier, one manufacturer and one contractor with risk aversion considered in different scenarios. The manufacturer procures materials from the supplier, outsources his carbon-reduction business to a contractor and produces low-carbon product for end customers. The third-party contractor specializes in reducing carbon emissions for the manufacturer based on the revenue paid by its employer. With the Mean-Variance model, we develop a game model to analyze the risk-averse supply chain. By comparing the risk-neutral case with the risk-averse, we explore the impacts of risk aversion on performances of the low-carbon supply chain. The risk aversion we have obtained does not affect the ratio of the manufacturer’s and the contractor’s profits, which shows its partnership to be based on a profit-sharing contract. The risk-aversion of chain members affects the supplier’s wholesale price decision, transforming from a self-focusing variable to a stretch-focus one. It is also affected by the positive impact of the risk aversion of its partners and is adversely influenced by its own risk aversion. This discipline also applies to retail price, payment level as well as manufacturer’s profit. We also show that the manufacturer should try to choose a risk-averse supplier as its partner if possible and strive to accept the demand risk in some extent to maximize its utility. However, the supplier may not necessarily follow the above rules, depending on the type of contractor used by the manufacturer. If the contractor is highly efficient in emission reduction, the supplier’s utility will deteriorate in the risk-aversion level of the manufacturer. On the contrary, the supplier will benefit from manufacturer’s risk aversion. In the end, we propose a contract to coordinate the decentralized supply chain via properly adjusting the risk-averse levels of the supplier and the manufacturer.

Our paper contributes theoretically in several ways. Firstly, we study the impact of risk aversion on supply chain performances to figure out the operational mechanism for a risk-reverse supply system. Secondly, we introduce and study the role of the expert contractor in reducing carbon emissions in supply chains, which is abstract but reflects the reality. Thirdly, in the risk-aversion context, we propose a contract to coordinate the focal low-carbon supply chain. Although the extant literature also coordinates directly the cost or revenue between the traditional chain members, what we directly adjust is the belief with regard to the risk-aversion level and the new understanding on it. Particularly, the extant relevant literature generally treats risk-aversion level as an exogenous parameter, whereas we take advantage of it as a controllable adjustment to the mean demand based on the Mean-Variance model.

There also exist some limitations in this study. The conclusions may not apply to other supply chain models such as the down-risk. Although we realize the importance of the contractor for a low-carbon supply chain, we do not explore the principal-agent problem between the manufacturer and the contractor as well as private information issues, for example the potential demand information owned by the manufacturer. Clarifying the above issues can better facilitate understanding of the operational mechanism within low-carbon supply chains in the future.

## Figures and Tables

**Table 1:** Notations.

Notation	Implication
$c_{s}$	Supplier’s production cost per unit
$c_{s}$	Manufacturer’s production cost per unit
α	Potential demand
γ	Consumer’s sensitivity parameters in the emission-reduction level
β	Consumer’s sensitivity parameters in the retail price
k	Investment coefficient of carbon-reduction
θ	Cost coefficient
λ	Risk-averse level
σ	Standard deviation of demand
e	Carbon-reduction level
p	Retail price
w	Wholesale price
ρ	Payment level per unit level of carbon-reduction

## Appendix A

The Hessian matrix of the utility with respect to the retail price and the carbon reduction level is

(A1) $$H_{cs}\left(p,e\right)=\left[\begin{array}{cc} -2\beta & \gamma \\ \gamma & -k \end{array}\right]$$

When $2\beta k-\gamma^{2}>0$, the matrix H(p,e) is negative definite since −2β<0. Then the utility function regarding the retail price and the carbon reduction level has the optimal value.

The first derivative of the supply chain utility with respect to the retail price is as follows.

(A2) $$\frac{\partial U_{cs}}{\partial p}=\alpha -2p\beta +\gamma e-\lambda_{cs}\sigma +\beta \left(c_{m}+c_{s}\right)=0$$

The above Equation (A2) can be restated as

(A3) $$p=\frac{1}{2\beta }[\alpha +\gamma e+\beta \left(c_{m}+c_{s}\right)-\lambda_{cs}\sigma ]$$

The first derivative of $U_{cs}$ with respect to the carbon reduction level follows

(A4) $$\frac{\partial U_{cs}}{\partial e}=\gamma \left(p-c_{m}-c_{s}\right)-e\;k=0$$

Accordingly, we have

(A5) $$e=\frac{\gamma }{k}\left(p-c_{m}-c_{s}\right)$$

From the formulas (A3) and (A5), we have the solutions of the retail price and the carbon reduction level.

(A6) $$p^{cs}=\frac{k\left(\alpha -\lambda_{cs}\sigma \right)+\left(k\beta -\gamma^{2}\right)\left(c_{m}+c_{s}\right)}{2k\beta -\gamma^{2}}$$

(A7) $$e^{cs}=\frac{\gamma \left[\alpha -\beta \left(c_{m}+c_{s}\right)-\lambda_{cs}\sigma \right]}{2k\beta -\gamma^{2}}$$
